# Global and regional estimates of clinical and economic burden of low back pain in high-income countries: a systematic review and meta-analysis

**DOI:** 10.3389/fpubh.2023.1098100

**Published:** 2023-06-09

**Authors:** Francis Fatoye, Tadesse Gebrye, Cormac G. Ryan, Ushotanefe Useh, Chidozie Mbada

**Affiliations:** ^1^Department of Health Professions, Manchester Metropolitan University, Manchester, United Kingdom; ^2^Lifestyle Diseases, Faculty of Health Sciences, North-West University, Potchefstroom, South Africa; ^3^Centre for Rehabilitation, School of Health and Life Sciences, Teesside University, Middleborough, United Kingdom

**Keywords:** high income countries, economic burden, clinical burden, LBP, cost

## Abstract

**Introduction:**

Low back pain (LBP) is a common health problem, and the leading cause of activity limitation and work absence among people of all ages and socioeconomic strata. This study aimed to analyse the clinical and economic burden of LBP in high income countries (HICs) via systematic review and meta-analysis.

**Methods:**

A literature search was carried out on PubMed, Medline, CINAHL, PsycINFO, AMED, and Scopus databases was from inception to March 15th, 2023. Studies that assessed the clinical and economic burden of LBP in HICs and published in English language were reviewed. The methodological quality of the included studies was assessed using the Newcastle-Ottawa quality assessment scale (NOS) for cohort studies. Two reviewers, using a predefined data extraction form, independently extracted data. Meta-analyses were conducted for clinical and economic outcomes.

**Results:**

The search identified 4,081 potentially relevant articles. Twenty-one studies that met the eligibility criteria were included and reviewed in this systematic review and meta-analysis. The included studies were from the regions of America (*n* = 5); Europe (*n* = 12), and the Western Pacific (*n* = 4). The average annual direct and indirect costs estimate per population for LBP ranged from € 2.3 billion to € 2.6 billion; and € 0.24 billion to $8.15 billion, respectively. In the random effects meta-analysis, the pooled annual rate of hospitalization for LBP was 3.2% (95% confidence interval 0.6%–5.7%). The pooled direct costs and total costs of LBP per patients were USD 9,231 (95% confidence interval −7,126.71–25,588.9) and USD 10,143.1 (95% confidence interval 6,083.59–14,202.6), respectively.

**Discussion:**

Low back pain led to high clinical and economic burden in HICs that varied significantly across the geographical contexts. The results of our analysis can be used by clinicians, and policymakers to better allocate resources for prevention and management strategies for LBP to improve health outcomes and reduce the substantial burden associated with the condition.

**Systematic review registration:**

https://www.crd.york.ac.uk/prospero/#recordDetails?, PROSPERO [CRD42020196335].

## Introduction

Low back pain (LBP) is a common health problem in people of all ages and socioeconomic strata (1). LBP occurs in high-income, middle, and low-income countries. It is the leading cause of activity limitation and work absence (2). Estimates of the 1-year incidence of a first-ever episode of LBP range between 6.3 and 15.4%, while estimates of the 1-year incidence of any episode of LBP range between 1.5 and 36% (3). The global point prevalence of LBP was 9.4% (95% CI 9.0–9.8), while the disability-adjusted life years (DALYs) due to the condition increased from 58.2 million in 1990 to 83.0 million in 2010 (4). LBP is one of the primary reasons that patients visit primary care physicians (5) and it represents the highest percentage of referrals and workload for physical therapy utilization (6, 7). For example, in the United States, LBP accounts for 2.5–3% of all physician visits (8). Furthermore, LBP is a major cause of hospitalization, for example, during 1990–2002 period a total of 7,240 LBP hospitalizations were identified among 5,061 (1.3%) Finnish military conscripts (9).

LBP constitutes a significant economic burden on the individual, caregivers and society (10, 11). The economic impact of LBP can be assessed from a number of different perspectives, including that of the patient, hospital, healthcare providers, third-party payer, government agency, and society (12). Regardless of who incurs the costs or who receives the benefits, societal perspective that incorporates direct and indirect costs (13). In context, direct costs are defined as costs for goods and services used in the diagnosis and treatment, and prevention of the problem in question (13). Further, rehabilitation and other medical consequences of LBP and all the private costs incurred by the patient and family are also included in direct costs. On the other hand, indirect costs include the value of the output that is lost because people are impaired from working, typical cost items in this category are costs for early retirement pensions caused by disability, short term absence from work, and premature death (14). The direct and indirect costs associated with LBP are among the highest for chronic health conditions mainly in terms of the significant number of workdays lost (10). In 2006, a review of total costs associated with LBP in the United States showed that it exceeds $100 billion per year (15). Among studies providing estimates of direct costs, the largest proportion of direct medical costs for LBP was spent on physical therapy (17%) and inpatient services (17%) (16). Overall, the clinical and economic burden of LBP are substantial when its prevention and management of LBP are suboptimal (16).

Many studies have investigated the clinical and economic burden of LBP in HICs (3, 10, 16, 17). The biggest challenge for aggregating the clinical and economic burden data is due to the studies adoption of different methodological designs. The sources of this methodological difference could be the size of the underlying populations, the treatments applied, differences in health care systems regarding access to health care, and the prices of the treatments (18). This is the first systematic review that that assessed the clinical and economic burden of LBP in HICs via meta-analysis.

## Methods

### Search protocol and registration

In this study, we used the Preferred Reporting Items for Systematic Reviews and Meta-Analysis guideline (19). A protocol for this systematic review was prospectively registered on PROSPERO and can be found at https://www.crd.york.ac.uk/prospero/#recordDetails? ID = CRD42020196335.

### Search strategy

A literature search using PubMed, Medline, CINAHL, PsycINFO, AMED, and Scopus databases with studies published from inception to March 15th, 2023. The following keywords were used in the search: Low back pain, hospitalization, cost of illness, absenteeism, ambulatory care, drug costs, emergency medical services, healthcare costs, nursing services, economics, physicians visit, clinical impact, utilization, burden of illness, cost, nursing cost (Appendix 1). These search terms were combined using conjunctions words “AND” or “OR”. Further, a manual search of reference sections of the included studies was also checked for additional studies. The search was performed one author (TG) and cross checked by another author (FF) to reduce the presence of bias in the selection and exclusion of studies.

### Inclusion and exclusion criteria

This review involved original research conducted among patients with LBP in HICs that reported findings related to costs (direct and indirect). The World Bank's definition of high come country was adopted. Eligible studies included observational (cross-sectional or surveys), randomized controlled trials (RCTs) and modeling analyses of patients with LBP in hospitals, primary healthcare clinics and home care contexts that were published in peer-reviewed journals. Language filter was applied to delimit the search to studies published in English language only. Review articles, editorials, letters to the editor, news reports, conference abstracts, comments, as well as the results of dissertations were excluded in this review.

### Study selection and assessment of methodological quality

After selection of the articles in each database, duplicate articles were removed electronically: https://access.clarivate.com/login?app=endnote and manually. Following the removal of duplicates, titles and abstracts were screened independently by two reviewers (FF & TG) to identify eligible studies. The full texts of the identified studies were checked against the inclusion and exclusion criteria. When there was disagreement, it was addressed through consultation with the third reviewer (CM). Having retrieved the full text of studies that met the inclusion criteria, they were assessed for methodological quality using the Newcastle–Ottawa quality assessment scale (NOS) for cohort studies (20). The NOS contains nine items, categorized into three dimensions including selection and comparability. Studies were scored using a scale with a possible maximum of nine points where a score ≥ 6 indicated high-quality studies, a score between 3 and 6 as moderate and a score ≤ 3 as low quality (20).

### Data extraction

Data were extracted by two independent review authors (TG & FF). The following information was extracted for each study: authors, country and year of publication, study objective, data source, inclusion criteria, LBP definition, population characteristics (size, % male, and mean age), hospitalization, emergency department visit, physician visits, average total annual cost per patient and annual population cost. The cost per population indicates the annual costs estimate of the disease in a specific country. A summary table was used to display the extracted data.

### Data synthesis

A weighting procedure regarding the clinical and economic burden of LBP of the included studies was applied only when combining data from multiple studies was satisfied. Meta-analyses were undertaken using Comprehensive Meta-analysis software (Biostat, Inc., New Jersey, USA) version 3 for Windows, to determine the pooled clinical and economic burden of LBP in HICs. The random-effects method was used to provide more confident data considering the heterogeneity within and between reports.

All costs were converted from local currencies to United States Dollar using purchasing power parities (21). We adjusted the cost data to the reference year of March 2022 using consumer price index from the World Bank Website (22). This methodology is useful for cost of illness studies to reach better comparability between the different currencies (23).

## Results

### Included studies

**Figure 1:**
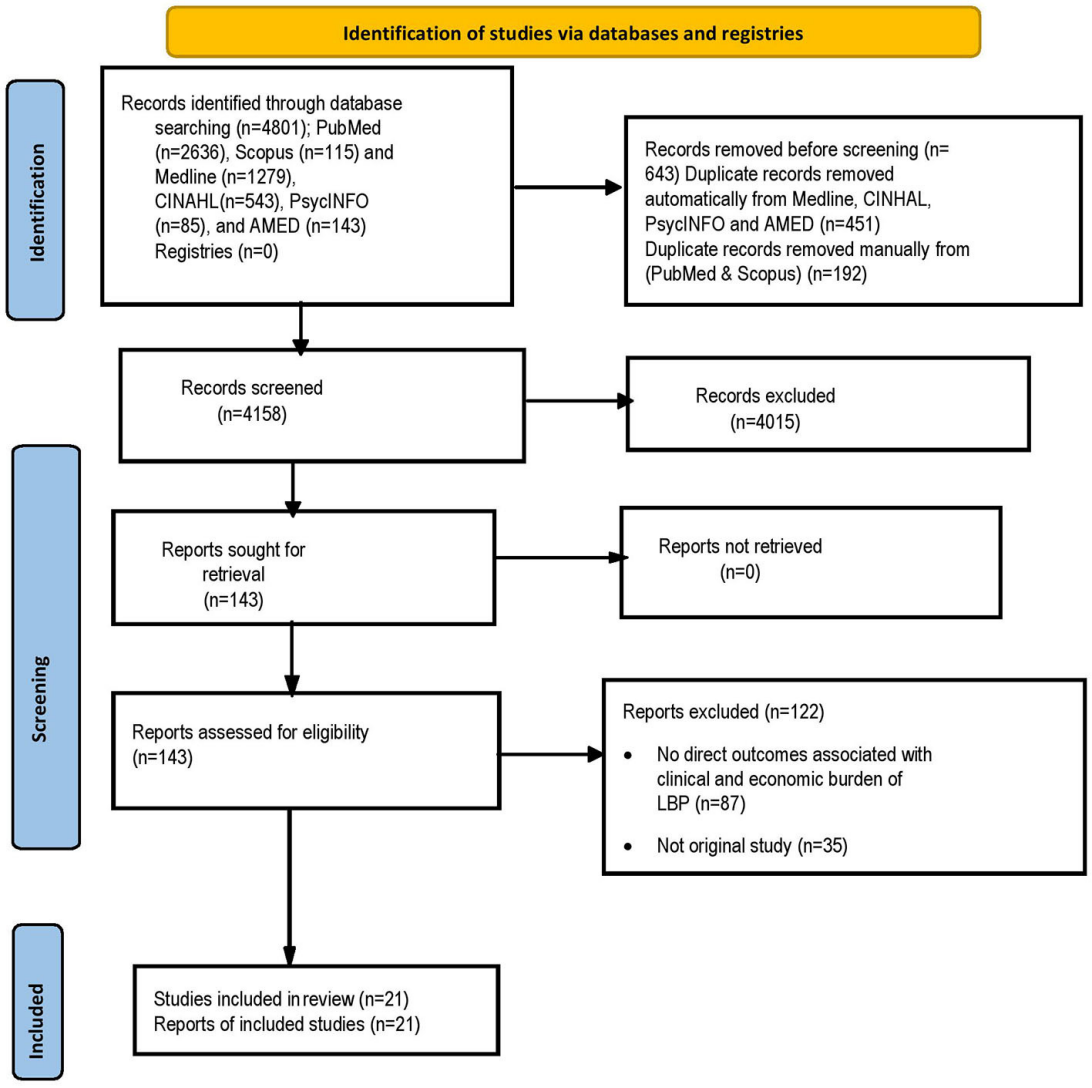
Flow diagram of publications included and excluded in the review.

The literature search identified 4,801 potentially relevant articles in PubMed (*n* = 2,636), Scopus (*n* = 115) and Medline (n = 1,279), CINHAL (*n* = 543), PsycINFO (*n* = 85), and AMED (*n* = 143) (Figure 1). Of these, 643 were duplicates. After screening the titles and abstracts 4,015 publications were excluded, leaving 143 articles for further full text review. Twenty-one studies met the inclusion criteria and were included in the review. The majority of studies included in this systematic review were of moderate to high quality based on the NOS score (Appendix 2). A further updated search yield two new articles that met the inclusion criteria (out of 1,230 identified potentially relevant articles).

### Characteristics of the included studies

Characteristics of the included studies are summarized and presented in Table 1. Of the 21 articles included, eight reported the clinical burden of LBP (8, 9, 28, 35, 38–40). Whereas, the remaining 13 studies reported the costs of LBP (11, 17, 24–26, 29–34, 36, 37). The included studies were conducted in United States, Spain, Switzerland, France, Finland, Japan, Netherland, Germany, United Kingdom, Sweden, and Australia (Table 1). According to the World Health Organization (WHO) classifications the included studies were from the regions of America (*n* = 5); Europe (*n* = 12) and the Western Pacific (*n* = 4).

**Table 1:** Characteristics of included studies.

**References/country/study period**	**Data source**	**Study objective**	**Inclusion criteria**	**LBP definition**	**WHO regions**
Alonso-García and Sarría-Santamera (24)/Spain	The National Health Survey of 2017 (NHS 2017)	To estimate the costs attributable to LBP, from a societal perspective	#Spanish population > 14 years of age #Have you suffered of LBP in the last 12 months?	N/A	EURO
Mattila et al. (9)/1990–2002/Finland/1990–1994	National Hospital Discharge Register	To investigate the incidence and trends of LBP hospitalization among Finnish military conscripts.	#All persons who started their military service during the study period. # The overall median length of hospital stay was three (range 1–114) days	ICD	EURO
Wieser et al. (25)/Switzerland/2005	Survey	To estimate the total cost of LBP in Switzerland in 2005 from a societal perspective	>18 years of age	Patient-reported	EURO
Depont et al. (26)/France/October 2001 and December 2002.	A structured questionnaire	To determine the total direct medical and non-medical cost of chronic low back pain (LBP)	#Aged 35–75 years #Have LBP at least once a week for at least 3 months #Consent to participate.	Patient-reported	EURO
Mattila et al. (27)/Finland/1979 to 1997	Adolescent Health and Lifestyle Survey	To investigate whether health, physical activity and other health behaviors, socio-demographic background and school success predict LBP hospitalization until early middle age.	Aged 14–18 years participating in a population survey between 1979 and 1997 was followed for an average of 11.1 years	ICD-10 codes	EURO
Leino-Arjas et al. (28)/Finland/1995	The Hospital Discharge Register	To assess the variation in hospital admission rates for back disorders by industry and occupational title among gainfully employed Finns.	Aged 25–64 years and occupationally active during the last week of 1995 (914,750 men and 868,886 women)	ICD-10 codes	EURO
Itoh et al. (29)/Japan	The review of public statistics and literature data	To assess the annual medical cost of work-related LBP in Japan.	Aged between 18 and 65 years, who visited any of over 30 Rosai Hospitals across Japan due to LBP	M45-M49, M50-M51 and M54.3-M54.5	WPRO
Gore et al. (17)/United States/2007 and 2008	LifeLink Health Plan Claims Database	To examine the comorbidities, treatment patterns, health care resource utilization, and direct medical costs of patients with chronic low back pain (CLBP) in clinical practice.	Patients with CLBP	ICD	PAHO
Itz et al. (30)/Netherlands/1 July 2008 and 1 January 2009	Data were retrieved from the Diagnosis Treatment Combination (DTC) registry of Vektis.	To examine the organization of medical specialist care and hospital costs for LBP	Patients referred to the hospital for LBP for the first time. DTCs related to rehabilitation medicine were excluded	N/A	EURO
Becker et al., (31)/Germany	This study is a secondary analysis of a 3-armed randomized controlled guideline implementation study in primary care	To describe the costs of care for patients with LBP	Age above 19, ability to read and understand German. Patients with pregnancy and isolated thoracic or cervical pain were excluded.	N/A	EURO
Hong et al. (32)/UK/January 1, 2007, to December 31,2009	The UK General Practice Research Database.	To assess 12-month health care costs associated with the treatment of CLBP.	Aged 18 years or older. Patients with non-organic psychoses were excluded	ICD, Ninth Revision, Clinical Modification (ICD-9-CM)	EURO
Olafsson et al. (11)/Sweden/2008–2011	Six Swedish national and regional registries	To estimate the societal costs of LBP.	Patients who underwent surgery anywhere in Sweden during 2000–2012 as registered in Swespine.	ICD-10 codes	EURO
Licciardone (8)/United States/2003–2004	The National Ambulatory Medical Care Survey	To elucidate the epidemiology and medical management of LBP during ambulatory medical care visits in the United States.	Physicians who met the criteria of being: (1) office-based; (2) principally engaged in patient care activities; (3) non-federally employed; and (4) not in the specialties of anesthesiology, pathology, or radiology.	Patient-reported	PAHO
Ekman et al. (33)/Sweden/2002	Cross-sectional data (questionnaire)	To analyze the health care resource use, productivity loss, and health-related quality of life of patients with CLBP	Age 18 years or older, had experienced LBP at least 50% of the days during the last 3 months. Patients with CLBP as a result of acute fractures, tumors, infection, or pregnancy were excluded	Patient-reported	EURO
Ivanova et al. (34)/US/1999 to 2006	Privately insured claims database	To assess the actual practice patterns of imaging, non-invasive therapy, medication use, and surgery in patients with LBP, and compare their costs to those of matched controls without LBP.	Aged 18– 64 years who had at least one LBP diagnosis, #Patients were required to have at least 6 months of continuous benefit eligibility before the initial LBP diagnosis.	ICD, Ninth Edition	PAHO
Taylor et al. (35)/United States/1987-1992	Washington State automated database	To examine recent trends and geographic variation of hospitalization in low back pain		ICD, Version 9	PAHO
Walker et al. (36)/Australia/2001	Survey	To estimate the cost-of-illness of LBP	18 years or older	ICD-10 codes	WPRO
van der Wurf et al. (37)/Netherlands/2015 to 2017	Occupational health services database in the Netherlands	To investigate the costs of LBP associated sick leave of workers in the Netherlands.	We included workers who were registered in the database with LBP associated sick leave episode.	ICD-10 codes	EURO
Hart et al. (38)/United States/three time periods (1980–81, 1985, 1989–90).	The National Ambulatory Medical Care Survey	To characterize the frequency of office visits for LBP/the contempt of ambulatory care, and how these vary by physician specialty.	It included nearly 3,000 office-based physician respondents not employed by the federal government in the 1990 survey.	ICD-9-CM codes	PAHO
Ferreira et al. (39)/Australia/2016–2019	Retrospective analysis	To investigate and quantify the extent of clinical variation in hospital admission following an ED presentation for LBP.	All ED presentations of patients aged between 18 and 111 with a discharge diagnosis of LBP with or without neurological signs and symptoms.	ICD-10, ICD-9 and SNOMED codes	WPRO
Buchbinder et al. (40)/Australia/2015	Retrospective review	To describe the characteristics and management of patients who presented to an Australian private hospital ED in 2015 with LBP	A triage presenting complaint code of either LBP; an ED discharge diagnosis of LBP or possibly related to LBP; and a main complaint of LBP in triage notes.	NA	WPRO

ICD, The International Classification of Diseases; PAHO, Region of the Americas; WPRO, Western Pacific Region; EURO, European Region; N/A, not available; ED, Emergency department; SNOMED, Systematized Nomenclature of Medicine Clinical Terms codes.

### Clinical burden

Table 2 provides an overview of hospitalization rates, physician visits and ambulatory visits. Of the six articles meeting the inclusion criteria, six contained hospitalization data (9, 28, 35, 39–41) and two had information on physician visit (8) and ambulatory visits (38) for LBP. Reported hospitalization rates, physician visits and ambulatory visits for LBP varied widely according to geographic region. In Finland, a total of 1.3% LBP hospitalization was reported from a population of military conscripts, the event-based incidence of LBP hospitalization was 27.0 per 1,000 person-years (9). Of the occupationally active Finns, 0.4% were hospitalized for LBP in 1996 (8). Further, hospitalization rates of 5.1 per 10,000 (35) and 1.1 per 100 (27) were reported in United States of America and Finland. Regarding ambulatory care or physician visits in the United States, LBP accounted for 2.8% (8) and 3% (38) during 2003–2004 and 1989–1990, respectively.

**Table 2:** Annual rate of hospitalizations, physician visit and ambulatory visit for LBP patients.

**References**	**Number of patients**	**Hospitalization, physician visits & ambulatory visits**
Mattila et al. (9)	387,070	#The proportion of conscripts hospitalized during military service due to LBP was 1.3%. #The event-based incidence of LBP hospitalization was 27.0 (95% CI: 25.7–28.2) per 1,000 person-years. #The incidence of hospitalization due to unspecified LBP was 19.1 per 1,000 person-years (95% CI: 18.3–20.4)
Leino-Arjas et al. (28)	7,253	Ratio of hospitalization = 3,124/7,253 (0.43)
Taylor et al. (35)	N/A	#In contrast, nonsurgical hospitalization rates fell from 15.5 to 5.1 per 10,000. #The proportion of operations involving fusion decreased from 15.8% in 1987 to 11.7% in 1990, and then remained stable
Mattila et al. (27)	57,408	#Hospitalization was 1.1%, #Incidence of 100 (95% CI: 94–108) per 100,000 person-years #The median length of hospital stay was 2 days (range 1–369)
Licciardone (8)	50,558	3.0% physician visit for LBP
Hart et al. (38)	15 million office visits for “mechanical” LBP in 1990	#The total number of LBP patients increased from 12,150,700 (1980 to 81) to 14,964,900 (1989 to 90) #All adult ambulatory visits = 2.8%.
Ferreira et al. (39)	176,729 LBP presentations to public hospital ED	#There were 44,459 hospital admissions from 2016–2019 #Unadjusted hospital admission rate of (25.5).
Buchbinder et al. (40)	450 LBP presentations to public hospital ED	# 238 (52.9%) were admitted to hospital

N/A, not available; ED, emergency department.

### Patient-level costs

Six studies reported patient-level direct and indirect costs for LBP (Table 3). The average direct cost estimate of LBP during 6 months were USD 959.43 (26) and USD 1,236.99 (31) in France and Germany, respectively. Average annual direct cost estimates in the general population ranged from USD 4,671.13 (9) to USD 10,430.20 (17) per LBP patient. Annual indirect costs, mainly productivity loss because of lost workdays of USD 26,579.57 per patient were reported for LBP in Sweden (33).

**Table 3:** Summary of studies that reported patient-level total direct costs for LBP.

**References**	**Sample, age**	**Costs per patient**	**Inflated 2022 $US**
Depont et al. (26)	796, 53 ± 11.3 years	DC = 715.6 € (95% CI: 644.2–797.8) over 6 months	USD 959.43 (95% CI: 864.43–1,069.74)
Becker et al. (31)	1,378, 48.73 ± 6.63 years	DC = 853.81 (713.6–1,044.7) TC = 1,789.81 (1,470.0–2,202.0) (over 6 months)	DC = USD 1,236.99 (95% CI 1,034.25–1,514.17) TC = 2,592.88 (95% CI 2,130.55–3,191.47)
Hong et al. (32)	64,167	TC = £1,074 ($1,681) per year	USD 1,643.66 per year
Ekman et al. (33)	302, 48.9 (14.2)	DC = 3,089 Euros (95% CI 2,208–3,971) per patient^¥^ TC = 20,666 Euros (95% CI 18,360–22,972) per patient per year^¥^	DC = USD 4,671.13 (95% CI 3,338.9–6,004.8) per year TC = 31,250.7 (95% CI 27,763.68–34,737.8) per year
Ivanova et al. (34)	5,211,551	DC = USD 7,211 ($18,695) per year	DC = USD 9,128.6 (23,666.43) per year
Gore et al. (17)	101,294, 47.2 ± 11.6 years.	Direct medical costs = USD 8,386 ± 17, 507 per year	Direct medical costs = USD 10,430.20 ± $21,774.6 per year

^¥^In 2002 prices; TC, total costs; DC, direct costs; IC, indirect costs.

### Population-level costs

Seven studies reported population level direct and indirect costs of LBP (Table 4). The average annual direct costs estimate per population for LBP ranged from USD 3.4 billion (29) to USD 3.6 billion (25). The included studies have also reported annual indirect costs per population for LBP ranged from USD 3.2 million (30) to USD 13.2 billion (36). A total cost of USD 14.45 and 12.2 billion for public hospitals and private hospitals was reported in the budget year 1999–2000 in Australia (36). The total economic burden of LBP in Sweden including all LBP episodes in 2011 was estimated at USD 967.3 million (11).

**Table 4:** Summary of studies that reported population-level costs for LBP.

**References**	**Number, age**	**Total annual population cost**	**Inflated 2022 $US**
Alonso-García and Sarría-Santamera (24)	8.16 million, >4 years	DC = 2.3 billion euros IC = 6.7 billion euro TC = 8.9 billion euro	DC = 3.4 billion IC = 9.9 billion TC = 13.4 billion million
Wieser et al. (25)	2,507, >18 years	DC = € 2.6 billion IC = € 4.1 billion	DC = 3.6 billion IC = 4.9 billion
Itz et al. (30)	80,652 (56%female), >50 years (61%)	TC = € 194 million Costs per patient = € 2410	TC = 249,531,488.9
Itoh et al. (29)	9,789, 20–64 years	Medical cost = 82.14 billion yen	Medical cost = 1 billion
Olafsson et al. (11)	129,97, 52.6 years	TC = € 740 million	TC = USD967.3 million
Walker et al. (36)	13.5 million adult (>18 years).	TC; AU$ 8.9 billion (public hospital); AU$ 7.5 billion (private hospital) DC = AU$ 1.02 billion IC = AU$ 8.15 billion	TC = 14.45 billion (public hospital) and 12.2 billion (private hospital) DC = 1.65 billion IC = 13.2 billion
van der Wurf et al. (37)	7,901	Total extrapolated sick leave costs € 244.7 million	Total extrapolated sick leave costs 3.2 million

TC, total costs; DC, direct costs; IC = indirect costs.

### Meta-analysis

**Figure 2:**
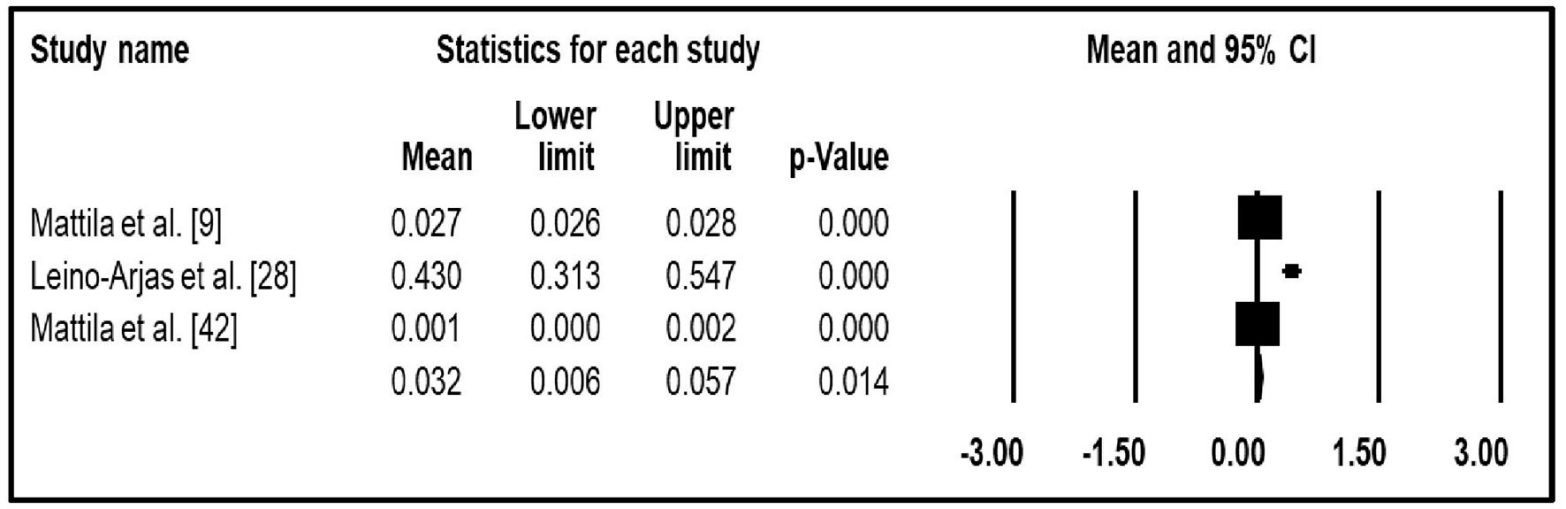
The pooled mean of annual rate of hospitalization of LBP.

Three studies that reported hospitalization data were quantitatively synthesized for meta-analysis (9, 23, 30). In the random effects meta-analysis, the pooled annual rate of hospitalization for LBP was 3.2% (95% CI: 0.6–5.7%) (Figure 2). The pooled direct costs (31, 33, 34) and total costs (26, 31, 33) of LBP per patients were USD 9,231 (95% CI −7,126.71 to 25,588.9) and USD 10,143.1 (95% CI 6,083.59–14,202.6), respectively.

## Discussion

This is the first systematic review and meta-analysis to assess the clinical and economic burden of LBP in HICs. The findings of the included studies varied substantially across the studies and countries. Our findings suggest that LBP is associated with a prolonged hospital length of stay, physician visit and ambulatory care. The meta-analysis, which derives from many patients, found that the rate of hospitalization, direct costs and total costs were 3.2% (95% CI 0.6–5.7%), USD 9,231 (95% CI −7,126.71–25,588.9) and USD 10,143.1 (95% CI 6,083.59–14,202.6), respectively.

The finding of the current study is in line with other studies (10, 16, 17) that assessed the economic impact of LBP. In those studies that reported the total costs, the indirect costs associated with LBP were higher than direct costs. Indirect costs in Spain, for example, represented 74.5% of the total costs of LBP (24). According to Alonso-García and Sarría-Santamera (24) the contributing factor to the high indirect costs of LBP was absenteeism and presenteeism. On the other hand, a cost-of-illness study in Australia reported that the costs of LBP in public hospitals was higher than in private hospitals (36). The high costs of LBP in public hospitals in Australia may be due to the universal health system, it provides medical, and hospitals cares for persons incapacitated with illness or injury including low back pain.

A total of six studies were included in this systematic review that reported the clinical burden of LBP in HICs. The reviewed literature suggested that the substantial clinical burden was reflected by high annual rate of hospitalisations, physician visits and ambulatory visits. In Finland, 1.3% annual rate of hospitalization was reported for LBP among military conscripts, this is much higher compared to the one reported in 1996 among 25- to 64-year-olds (9, 28). The annual rate of hospitalization for LBP in the current review are lower than other types of health conditions such as asthma where the overall rate of asthma hospitalization was 42 per 1,000 (41).

The key findings of this study confirm that LBP is associated with high clinical and economic burden in HICs. The review also revealed that the findings of the included studies varied significantly in terms of geographical location. The contributing factors to the differences of clinical and economic burden of LBP across the geographic areas could be the health system, health financing system, and sociodemographic characteristics of the people. The results of our analysis can be used by clinicians, and policymakers to better allocate resources for prevention and management strategies for LBP to improve health outcomes and reduce the substantial burden associated with the condition. We also hope our results will be of use to researchers planning to evaluate the cost-effectiveness of various strategies for preventing LBP in HICs.

There are a number of strengths and limitations of this study that need to be considered. The main strength of this review is the comprehensiveness of the search terms, screening of numerous data bases, and assessment of methodological quality of the studies. Only studies published in English language were included. Therefore, it is possible that relevant studies published in other languages may have been excluded. We did not use back pain as a search term, this is because “low back pain” is the key term used primarily in the literature and major international studies such as the global burden of disease study. Further, reported clinical and economic burden of LBP in HICs are limited by a large heterogeneity of available data. In spite of these limitations, we believe that this review was systematic in nature and summarizes all available and relevant clinical and economic burden results from the literature.

## Conclusion

LBP leads to high clinical and economic burden in HICs that varies significantly across the geographical contexts. We also found that LBP is a common hospital-associated problem with a clear impact on length of stay and hospital costs. Knowledge of the clinical and economic impact of LBP in HICs is useful to influence programs and behavior in healthcare facilities, to guide policy makers and funding agencies to improve the health outcomes of individuals with the condition and reduce its huge economic burden.

## Data availability statement

The original contributions presented in the study are included in the article/Supplementary material, further inquiries can be directed to the corresponding author.

## Author contributions

TG and FF collected the data. All authors made substantial contributions to conception and design of the study. All authors interpreted the data, revised the draft critically, and approved the submitted manuscript.
