# Supplementary material for: Global and regional estimates of clinical and economic burden of low back pain in high-income countries: a systematic review and meta-analysis
Source: Front Public Health. 2023 Jun 9;11:1098100. doi: 10.3389/fpubh.2023.1098100 (PMC10298167; doi:10.3389/fpubh.2023.1098100)
Supplement: Supplementary file 1 [file Data_Sheet_1.docx]

**Appendix 1: Clinical and economic burden search strategy**

**Appendix 1a:** Medline, CINAHL, PsycINFO and AMED (inception to 16th March/2023

| Search ID | Search Terms |
| --- | --- |
| S23 | S21 AND S22 |
| S22 | S1 OR S2 OR S3 OR S4 OR S5 OR S6 OR S7 OR S8 OR S9 OR S10 OR S11 OR S12 OR S13 OR S14 OR S15 OR S16 OR S17 OR S18 OR S19 OR S20 |
| S21 | AB low back pain |
| S20 | AB hospitalization |
| S19 | AB cost of illness |
| S18 | AB absenteeism |
| S17 | AB ambulatory care |
| S16 | AB drug cost |
| S15 | AB emergency medical services |
| S14 | AB healthcare services |
| S13 | AB nursing services |
| S12 | AB economics |
| S11 | AB physician |
| S10 | AB burden |
| S9 | AB clinical impact |
| S8 | AB utilization |
| S7 | AB burden of illness |
| S6 | AB cost |
| S5 | AB nursing costs |
| S4 | AB physician cost |
| S3 | AB physician visits |
| S2 | AB emergency department visits |
| S1 | AB years lived with disability |

**Appendix 1b:** Scopus (inception to 16th March/2023)

| Search ID | Search Terms |
| --- | --- |
| 1 | TITLE-ABS-KEY ( low AND back AND pain ) |
| 2 | TITLE-ABS-KEY ( hospitalisation ) |
| 3 | TITLE-ABS-KEY ( cost AND of AND illness ) |
| 4 | TITLE-ABS-KEY ( absenteeism ) |
| 5 | TITLE-ABS-KEY ( ambulatory AND care ) |
| 6 | TITLE-ABS-KEY ( drug AND costs ) |
| 7 | TITLE-ABS-KEY ( emergency AND medical AND services ) |
| 8 | TITLE-ABS-KEY ( healthcare AND costs ) |
| 9 | TITLE-ABS-KEY ( nursing AND services ) |
| 10 | TITLE-ABS-KEY ( economics ) |
| 11 | TITLE-ABS-KEY ( physicians ) |
| 12 | TITLE-ABS-KEY ( burden ) |
| 13 | TITLE-ABS-KEY ( clinical AND impact ) |
| 14 | TITLE-ABS-KEY ( utilization ) |
| 15 | TITLE-ABS-KEY ( burden AND of AND illness ) |
| 16 | TITLE-ABS-KEY ( cost ) |
| 17 | TITLE-ABS-KEY ( nursing AND cost ) |
| 18 | TITLE-ABS-KEY ( physician AND cost ) |
| 19 | TITLE-ABS-KEY ( physician AND visit ) |
| 20 | ( TITLE-ABS-KEY ( hospitalisation ) ) OR ( TITLE-ABS-KEY ( cost AND of AND illness ) ) OR ( TITLE-ABS-KEY ( absenteeism ) ) OR ( TITLE-ABS-KEY ( ambulatory AND care ) ) OR ( TITLE-ABS-KEY ( drug AND costs ) ) OR ( TITLE-ABS-KEY ( emergency AND medical AND services ) ) OR ( TITLE-ABS-KEY ( healthcare AND costs ) ) OR ( TITLE-ABS-KEY ( nursing AND services ) ) OR ( TITLE-ABS-KEY ( economics ) ) OR ( TITLE-ABS-KEY ( physicians ) ) OR ( TITLE-ABS-KEY ( burden ) ) OR ( TITLE-ABS-KEY ( clinical AND impact ) ) OR ( TITLE-ABS-KEY ( utilization ) ) OR ( TITLE-ABS-KEY ( burden AND of AND illness ) ) OR ( TITLE-ABS-KEY ( cost ) ) OR ( TITLE-ABS-KEY ( nursing AND cost ) ) OR ( TITLE-ABS-KEY ( physician AND cost ) ) OR ( TITLE-ABS-KEY ( physician AND visit ) ) |
| 21 | ( TITLE-ABS-KEY ( low AND back AND pain ) ) AND ( ( TITLE-ABS-KEY ( hospitalisation ) ) OR ( TITLE-ABS-KEY ( cost AND of AND illness ) ) OR ( TITLE-ABS-KEY ( absenteeism ) ) OR ( TITLE-ABS-KEY ( ambulatory AND care ) ) OR ( TITLE-ABS-KEY ( drug AND costs ) ) OR ( TITLE-ABS-KEY ( emergency AND medical AND services ) ) OR ( TITLE-ABS-KEY ( healthcare AND costs ) ) OR ( TITLE-ABS-KEY ( nursing AND services ) ) OR ( TITLE-ABS-KEY ( economics ) ) OR ( TITLE-ABS-KEY ( physicians ) ) OR ( TITLE-ABS-KEY ( burden ) ) OR ( TITLE-ABS-KEY ( clinical AND impact ) ) OR ( TITLE-ABS-KEY ( utilization ) ) OR ( TITLE-ABS-KEY ( burden AND of AND illness ) ) OR ( TITLE-ABS-KEY ( cost ) ) OR ( TITLE-ABS-KEY ( nursing AND cost ) ) OR ( TITLE-ABS-KEY ( physician AND cost ) ) OR ( TITLE-ABS-KEY ( physician AND visit ) ) ) |

**Appendix 1c:** PubMed (inception to 16th March /2023)

| Search terms |
| --- |
| (((((((((((((hospitalisation[MeSH Terms])) OR (Cost of illness[MeSH Terms]))) OR (healthcare costs[MeSH Terms]))) OR (drug costs[MeSH Terms]))) OR (Absenteeism[MeSH Terms]))) OR (Ambulatory care[MeSH Terms]))) OR (Emergency medical services[MeSH Terms]))) OR (hospital nursing services[MeSH Terms]))) OR (Economics[MeSH Terms]))) OR (burden of illness[MeSH Terms]))) OR (Physicians[MeSH Terms]))) OR (allocation, cost[MeSH Terms]))) OR (emergency[MeSH Terms]))) AND (low back pain[MeSH Terms])) |
| low back pain[MeSH Terms] |
| emergency[MeSH Terms] |
| allocation, cost[MeSH Terms] |
| Physicians[MeSH Terms] |
| burden of illness[MeSH Terms] |
| Economics[MeSH Terms] |
| hospital nursing services[MeSH Terms] |
| Emergency medical services[MeSH Terms] |
| Ambulatory care[MeSH Terms] |
| Absenteeism[MeSH Terms] |
| drug costs[MeSH Terms] |
| healthcare costs[MeSH Terms] |
| Cost of illness[MeSH Terms] |
| hospitalisation[MeSH Terms] |

**Appendix 2: Study quality assessment**

**Appendix 2a:** Quality assessment checklist for nonrandomized studies.

| Domain | Checklist Criteria | Additional Information |
| --- | --- | --- |
| Selection | 1. Is the case definition adequate?    1. yes, with independent validation *    2. yes, eg record linkage or based on self-reports    3. no description |  |
|  | 1. Representativeness of the cases    1. consecutive or obviously representative series of cases *    2. potential for selection biases or not stated | Assumption: Given the nature of the review, studies received a star if they discuss representation (and reasons for their study being representative) or if they are multicentre/regional/national. |
|  | 1. Selection of Controls    1. community controls *    2. hospital controls    3. no description | Assumption: It was assumed that if the study was in a hospital setting in which cases were hospital patients, hospital controls were accepted. |
|  | 1. Definition of Controls    1. no history of disease (endpoint) *    2. no description of source | Assumption: History of disease/infection was used in this criteria even in studies looking at mortality or other burden outcomes |
| Comparability | 1) Comparability of cases and controls on the basis of the design or analysis   1. study controls for age/sex/comorbidities * 2. study controls for any additional factor * | 2 * maximum allotted for this criteria.  Assumption: For studies in which hospital associated cases and LoS were being analysed, two stars were only given if time dependency was controlled for |
| Exposure | 1. Ascertainment of exposure    1. secure record (eg surgical records) *    2. structured interview where blind to case/control status *    3. interview not blinded to case/control status    4. written self-report or medical record only    5. no description | Assumption: Studies which utilised lab techniques were used to ascertain exposure received one star. |
|  | 1. Same method of ascertainment for cases and controls    1. yes *    2. no |  |
|  | 1. Non-Response rate    1. same rate for both groups *    2. non respondents described    3. rate different and no designation | Assumption: No description of data cleaning or linkage and loss to missing data for retrospective studies was panelised by not awarding a star |

**Appendix 2b:** Study quality of the included study.

| Study | Selection 1) * | Selection 2)* | Selection 3)* | Selection 4)* | Comparability 1)** | Exposure 1) * | Exposure 2)* | Exposure 3) * | Total (MAX. 9) |
| --- | --- | --- | --- | --- | --- | --- | --- | --- | --- |
| Alonso-García & Sarría-Santamera, [38] | 1 | 1 | 1 | 0 | 1 | 1 | 1 | 0 | 6 |
| Mattila et al. [9] | 1 | 1 | 1 | 0 | 2 | 1 | 1 | 0 | 7 |
| Wieser et al. [34] | 1 | 1 | 1 | 0 | 0 | 1 | 1 | 0 | 5 |
| Depont et al. [32] | 1 | 1 | 1 | 1 | 2 | 1 | 1 | 0 | 8 |
| Mattila et al. [40] | 1 | 1 | 1 | 0 | 0 | 1 | 1 | 0 | 7 |
| Leino-Arjas et al. [26] | 1 | 1 | 1 | 0 | 2 | 1 | 1 | 0 | 7 |
| Itoh et al. [36] | 1 | 1 | 1 | 0 | 0 | 1 | 1 | 0 | 5 |
| Gore et al. [17] | 1 | 1 | 1 | 1 | 1 | 1 | 1 | 0 | 7 |
| Itz et al. [37] | 1 | 1 | 1 | 0 | 0 | 1 | 1 | 0 | 5 |
| Becker et al., [31] | 1 | 1 | 1 | 0 | 1 | 1 | 1 | 0 | 6 |
| Hong et al. [35] | 1 | 1 | 1 | 0 | 0 | 1 | 1 | 0 | 5 |
| Olafsson et al. [11] | 1 | 1 | 1 | 1 | 1 | 1 | 1 | 0 | 7 |
| Ekman et al. [30] | 1 | 1 | 1 | 0 | 0 | 1 | 1 | 0 | 5 |
| Ivanova et al. [33] | 1 | 1 | 1 | 0 | 0 | 1 | 1 | 0 | 5 |
| Licciardone,[27] | 1 | 1 | 1 | 1 | 2 | 1 | 1 | 0 | 8 |
| Taylor et al. [24] | 1 | 1 | 1 | 0 | 2 | 1 | 1 | 0 | 7 |
| Walker et al. [29] | 1 | 1 | 1 | 0 | 0 | 1 | 1 | 0 | 5 |
| van der Wurf et al. [39] | 1 | 1 | 1 | 0 | 0 | 1 | 1 | 0 | 5 |
| Hart et al. [25] | 1 | 1 | 1 | 1 | 2 | 1 | 1 | 0 | 8 |
| Ferreira et al., 2022 | 1 | 1 | 1 | 1 | 1 | 1 | 1 | 0 | 7 |
| Buchbinder et al., 2022 | 1 | 1 | 1 | 0 | 0 | 1 | 1 | 0 | 5 |
